# Follicle-Stimulating Hormone Receptor Expression and Its Potential Application for Theranostics in Subtypes of Ovarian Tumors: A Systematic Review

**DOI:** 10.3390/cancers16061140

**Published:** 2024-03-13

**Authors:** Marie-Christine E. Bakker, Geertruid J. Brink, Alex J. Poot, Arthur J. A. T. Braat, Geertruida N. Jonges, Ronald P. Zweemer

**Affiliations:** 1 Department of Gynaecological Oncology, Cancer Center, University Medical Center Utrecht, 3584 CX Utrecht, The Netherlands; m.e.bakker2@students.uu.nl (M.-C.E.B.); g.j.brink-7@umcutrecht.nl (G.J.B.); 2 Department of Radiology and Nuclear Medicine, University Medical Center Utrecht, 3584 CX Utrecht, The Netherlands; a.j.poot@umcutrecht.nl (A.J.P.);; 3 Department of Pathology, University Medical Center Utrecht, 3584 CX Utrecht, The Netherlands; g.n.jonges@umcutrecht.nl

**Keywords:** ovarian cancer, FSH receptor, theranostics

## Abstract

**Simple Summary:**

In the past decades, the survival of ovarian malignant tumors has been largely unchanged. Treatment usually consists of a combination of systemic and surgical therapy. However, when recurrence occurs, treatment options are limited. When repeated surgery is no longer possible and chemotherapy is no longer effective, theranostics, a newly developing approach, might be of value. Theranostics will diagnose and destroy malignant tissue by binding radioactive isotopes to targets on malignant cells. To develop this approach, selection and validation of a target is necessary. For the selection of a target in ovarian tumors, the follicle-stimulating hormone (FSH) receptor appears to be suitable because it is primarily expressed in ovarian tissue, and expression is reported in malignant tumors. This review seeks to determine whether the follicle-stimulating hormone receptor is expressed in ovarian tumor types and, therefore, is suitable as a target in a future theranostic approach.

**Abstract:**

Ovarian cancer mortality rates have not decreased significantly in the past years. As most women are still diagnosed in an advanced stage, there is a need for new treatment strategies for recurrent disease. A potentially new developing targeted approach, theranostics, combines diagnostics and treatment using radiopharmaceuticals. Through target receptors, imaging and treatment of malignant tissue can be achieved. For ovarian malignancy, the follicle-stimulating hormone (FSH) receptor may serve as a possible target since expression appears to be limited to ovarian cells. In this systematic review, we aim to gather all available literature on the expression of the FSH receptor in ovarian tumors. Pubmed, Embase and the Cochrane databases were searched until December 2023 for eligible studies. The search yielded 41 studies, mostly regarding serous carcinomas, sex cord–stromal tumors (SCSTs) and cell lines of serous and SCSTs. Various techniques were used to analyze the expression of the FSH receptor. For serous carcinomas, conflicting results on the expression of the FSH receptor were found. Studies on SCSTs, mainly studying the subtype of granulosa cell tumors, all showed positive expression of the FSH receptor. In the cell lines studies, the KGN cell line derived from a granulosa cell tumor shows positive expression in all studies. Available studies show that SCSTs express the FSH receptor. A theranostic approach targeting the FSH receptor may, therefore, provide a useful new approach for this malignancy with limited therapeutic options in recurrent disease.

## 1. Introduction

In 2020, over 300,000 new cases of cancer of the ovary were reported, and that same year, over 200,000 women died of the disease. Mortality rates are excessive since ovary tumors are often diagnosed at an advanced stage. Thereby, mortality rates have been largely unchanged over the last decades [1].

The ovaries are composed of different types of cells, from which, after carcinogenesis, several types of tumors may emerge. Hence, ovarian tumors are classified based primarily on their histopathological patterns [2,3]. Most ovarian cancers are surface-epithelial cell carcinomas, serous, mucinous or other types; a smaller group is derived from germ cells, and, in addition, 5–8% of the primary ovarian tumors are tumors originating from the sex cord or stromal cells [2].

Ovarian cancer treatment usually consists of a combination of systemic and surgical therapy. However, when recurrence occurs, treatment options are limited. When repeated surgery is no longer possible and chemotherapy is no longer effective, theranostics, a newly developing approach, might be valuable. Theranostics, combining therapeutics and diagnostics, will diagnose and destroy malignant tissue by binding a radioactive isotope to a targeting molecule to selectively bind the tumor cells. After binding, the isotope is internalized and able to identify or destroy the cell with the radioactivity from within [4].

This targeted radionuclide approach originated in the treatment of benign and malignant thyroid diseases. Nowadays, it is implemented for a whole range of malignancies, with it being available as a standard therapy, particularly for neuroendocrine tumors and prostate cancer [5,6,7,8]. A receptor, needed as a target, is preferably exclusively expressed on malignant cells to avoid damage to healthy tissue and, thereby, limit side effects. The difficulty in developing this therapy is the selection and validation of targets and the development of radiopharmaceuticals against these targets.

For the selection of a target in ovarian tumors, the follicle-stimulating hormone (FSH) receptor emerges as a promising target given its surface location as a G-protein coupled receptor with its characteristic 7-transmembrane-helix protein structure, crucial for combining extracellular and intracellular signal transduction [9]. Upon ligand binding on the surface, the isotopes used in theranostics should be internalized, capturing radioactivity within the cell for diagnostic (imaging) and therapeutic (irradiation) purposes rather than primarily instigating a pharmacological response [4]. Therefore, target expression and validation are required to develop a theranostic approach.

Furthermore, the exclusive expression of the FSH receptor in the reproduction system, particularly in ovarian tissue to mature follicles under the influence of FSH, along with its reported maintenance in ovarian cancers while being absent in other healthy tissue, underscores its attractive appearance as a therapeutic target [10,11,12,13,14]. This selectivity is favorable when treating this target since the side effects are minimized.

Although the FSH receptor has been studied before in other malignancies, including prostate, breast, colon, pancreas, urinary bladder, lung and testis carcinomas, in order to further develop theranostics focusing on ovarian tumors, a review on the expression of the FSH receptor specifically in ovarian tumors was performed [12,13,15,16].

## 2. Materials and Methods

A systematic literature search was conducted to gather all available literature concerning the FSH receptor. The PRISMA guidelines were followed throughout the process [17]. The systematic review is registered in the PROSPERO database (ID CRD42024508414).

Studies regarding all types of malignant ovarian tumors in patients, cell lines or animal studies examining the presence or absence of the FSH receptor were included. All possible detection methods for the presence of the FSH receptor were accepted since no consensus has been reached in regard to the gold standard of detecting the expression of the FSH receptor. Exclusion criteria were benign ovarian tumors, literature reviews, conference abstracts, comments on articles, non-English articles, and manuscripts reanalyzing original data of an included article or papers with a lack of original data.

The search terms included all types of ovarian cancer paired with the FSH receptor and were run through the Pubmed, Embase and Cochrane databases, as presented in Supplementary Text S1. The search in Embase was filtered on the source (Embase, Embase + Medline) and publication type (article, article in press). The databases were last consulted on the 31 December 2023, to verify all the articles obtained.

In all the publications that emerged from the search, duplications were identified throughout the use of Rayyan and manually reviewed and excluded [18]. Of the unique articles, the titles and abstract were screened first and, subsequently, full text by two authors independently (MB/GB).

From all the included articles, data were extracted regarding author, year of publication, study design (cohort study, case report, etc.), type of ovarian tumor, population (size of study population/tumor samples, human tumor samples/cell lines/animal tumor sample, stage of tumors, primary or recurrent tumor), analyze methods, FSH receptor expression and possible information regarding controls or receptor mutation status. Data from databases were not extracted when patient characteristics or methods of analysis were unclear.

From the included studies, the differing outcome measures were grouped for comparison: whether an article detected the FSH receptor (yes or no) with a binding assay or by detecting its RNA or DNA, for instance, or a mean percentage of tumors that expressed the FSH receptor, when an article used staining techniques.

Quality assessment tools per study design used were the JBI tool (descriptive cross-sectional studies) and the Murads tool (case reports) [19,20]. The quality of evidence was classified as low, moderate or good quality. All articles were blind-reviewed by the two authors (MB/GB), and scores were compared. When scores differed, articles were discussed, and a consensus was reached.

## 3. Results

### 3.1. Study Selection

**Figure 1:**
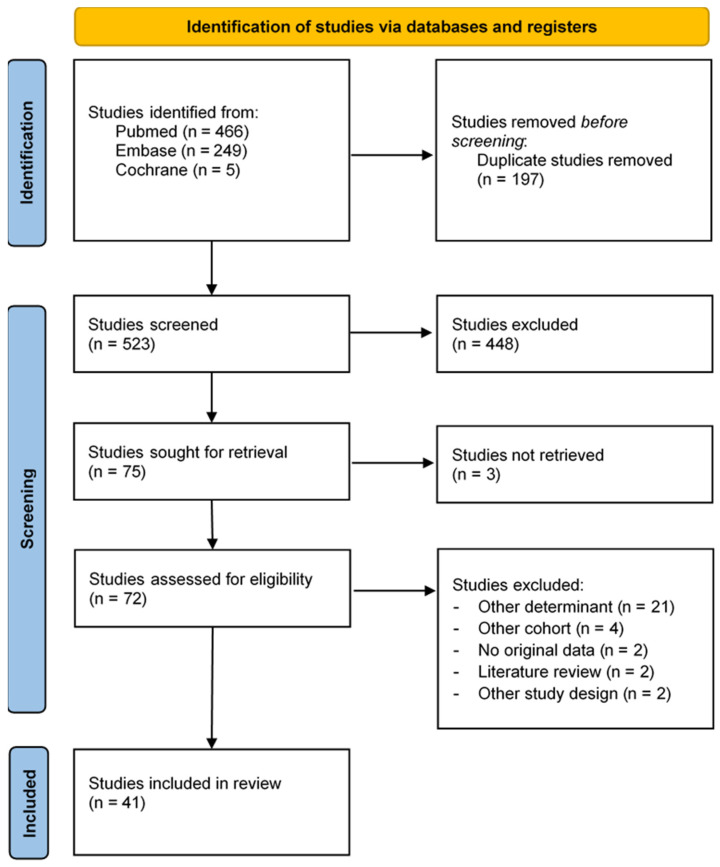
Flowchart of FSH receptor literature search (PRISMA flow diagram template 2020).

The literature search regarding the presence of the FSH receptor on all types of ovarian tumors yielded 523 unique articles on the Pubmed, Embase and Cochrane databases. After title and abstract screening, 448 studies were excluded; the remaining 72 were screened based on full text. Eventually, 41 articles remained for data extraction, as presented in the PRISMA flowchart in Figure 1 [17].

### 3.2. Study Characteristics

**Figure 2:**
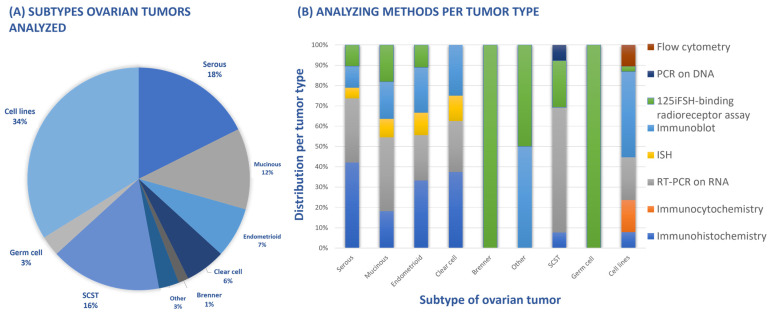
All types of ovarian tumors analyzed by various methods to detect FSH receptor expression. (**A**) Distribution of type of ovarian tumors examined by included studies (n = 41); (**B**) different techniques to examine the FSH receptor, distributed per type of ovarian tumor. SCST: sex cord–stromal tumor; RT: reversed transcriptase; PCR: polymerase chain reaction; ISH: in situ hybridization; iFSH: Iodium-labeled FSH.

As presented in Figure 2A, the majority of the 41 included studies conducted research on FSH receptor expression in cell lines (n = 23) or serous ovarian cancer (n = 12). In addition, sex cord–stromal tumors were highly studied as well (n = 11), followed by mucinous, endometrioid, clear cell, Brenner, germ cell and ‘other’ carcinoma consisting of a carcinosarcoma and an undifferentiated carcinoma.

Different methods were used to study the presence of the FSH receptor on malignant cells: immuno(histo)chemistry (IHC), where the cells were stained and scored utilizing the immunoreactive score (IRS), Real-Time Polymerase Chain Reaction (RT-PCR) to identify RNA or DNA of the receptor, in situ hybridization (ISH) to detect nucleotide sequences, immunoblot (Northern, Western) to recognize proteins by binding with an antibody, [^125^I]-FSH-binding radioreceptor assay and flow cytometry. In Figure 2B, an overview is presented of the different analyzing methods per tumor type. There were 12 studies reporting serous carcinoma, for instance, of which 7 used IHC as an analyzing method, 6 used RT-PCR on RNA, 1 ISH, 2 used immunoblots, and 2 were through the use of [^125^I]-FSH-binding radioreceptor assay. Of the eight studies conducting research on mucinous tumors, two used IHC, four used RT-PCR on RNA, one used ISH, two used immunoblots, and two used [^125^I]-FSH-binding radioreceptor assay. In addition, SCSTs were studied in 11 articles, of which 1 study used IHC, 8 used RT-PCR on RNA, 3 used [^125^I]-FSH-binding radioreceptor assay, and 1 used RT-PCR on DNA, as visualized in Figure 2B.

### 3.3. Risk of Bias in Studies

The results of the risk-of-bias assessment for all 41 studies, using the “Risk-of-bias VISualization” tool, are visualized in Supplementary Figure S1a,b [21]. The majority of included studies related to the FSH receptor were found to be descriptive cross-sectional studies; therefore, 41 articles were assessed using the JBI tool [19]. The majority of studies (38) were rated good quality with low risk of bias and 3 were rated moderate quality. The Murads tool was used for two case report studies, both marked as good quality [20]. No study was excluded based on risk-of-bias assessment.

### 3.4. FSH Receptor Expression

In order to evaluate the FSH receptor expression in the different types of ovarian tumors, the results were organized by tissue or cell line study, type of tumor and analyzing method. All studies reported their method of analysis for the FSH receptor expression. Different analyses were used to measure FSH receptor expression, showing varying outcomes. Some studies note the IRS score, some exclusively marked expression by yes or no, and others compare expression levels with control groups. The IRS is defined as the percentage of positive cells multiplied by the intensity of staining; the median FSH receptor expression level (IRS = 3) is often used with a cut-off to define FSH receptor positive (IRS ≥ 3) versus FSH receptor negative (IRS < 3) tumors. Results are presented in Table 1.

#### 3.4.1. Serous Carcinomas

**Figure 3:**
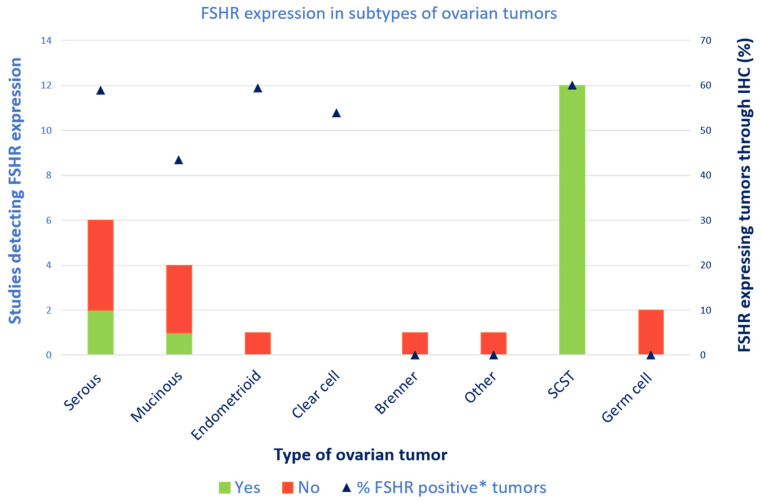
FSH receptor expression in subtypes of ovarian tumors, visualized by two outcomes: detection of FSH receptor by yes or no (left Y-axis, bar graph) and mean percentage of tumors expressing the FSH receptor using IHC (right Y-axis, triangular points). FSHR: follicle-stimulating hormone receptor; SCST: sex cord–stromal tumor; IHC: immunohistochemistry. * Immunoreactive score (IRS) > 3.

Of the 12 studies regarding serous carcinomas, 3 studies were specifically performed on high-grade serous carcinomas and 1 on low-grade serous carcinomas. Two studies detected expression of the FSH receptor [24,42], and four studies found little to no expression [22,25,28,30], as presented in Table 1 and Figure 3.

Of the six studies analyzing the FSH receptor using IHC, an average of 58.9% (44.9–84.0) of the tumors with an IRS score of >3 was found. Furthermore, five articles used RT-PCR, two used [^125^I]FSH-receptor-binding assays, and one used ISH and immunoblot, respectively, showing inconsistent results.

In addition to the presence or absence of FSH receptor expression, three studies suggested that FSH receptor expression is related to tumor stage or grade. Wang, Lin [42] compared differently graded tumors and found a decrease in FSH receptor expression during the loss of tumor cell differentiation. Even when analyzing heterogeneous areas within the same tumors, this loss was observed. Antral follicles served as positive controls, whereas the levels were compared with benign tumor specimens as well; the highest levels were found in serous borderline tumors, followed by serous cystadenomas, papillary serous cystadenomas and grade 1 carcinomas. Similarly, Garrido, Bruneau [24] found a significant decrease in FSH receptor mRNA expression in EOC stage III compared to stage I. Cheung, Lokman [22] found the FSH receptor expression to be reduced in HGSOC compared to benign ovarian tumors.

This is also observed in two studies investigating subgroups of serous carcinomas comparing high-grade and low-grade serous carcinomas. Feng, Wen [33] showed 84.0% of the 25 primary low-grade serous carcinomas to be FSH receptor-positive, significantly differing from high-grade tumors (54.9%). This was in line with the 44.9–55.3% staining intensity of the high-grade tumors in a different study by Feng et al. (2016) [31].

Overall, serous carcinomas show differing, inconsistent expression results of the FSH receptor using various analyzing techniques. Furthermore, FSH receptor expression was found to be related to tumor stage and grade.

#### 3.4.2. Mucinous Carcinomas

Mucinous carcinomas were examined in eight studies through differing techniques. RT-PCR was used in four studies, two used immunoblot, two used [^125^I]FSH-receptor-binding, and one used ISH, as presented in Table 1. Of the two studies analyzing the FSH receptor using IHC, an average of 43.5% (61.9–25.0) of the tumors with an IRS score of >3 was found [23,29].

One research article detected FSH receptor RNA using RT-PCR and found no mutations in the receptor genome of the tumor cells [34].

#### 3.4.3. Sex Cord–Stromal Tumors

Furthermore, 11 studies conducted research on sex cord–stromal tumors, of which 9 studies examined the adult-type granulosa cell tumor. Additionally, the Sertoli–Leydig cell tumor and a granulosa cell–theca cell tumor were studied by one paper and two papers, respectively. The majority of studies used RT-PCR to detect FSH receptor RNA in tumor cells. All studies of all types of sex cord–stromal tumors detected the FSH receptor.

By using IHC, one study reported 60% of 175 FFPE samples of 138 patients suffering granulosa cell tumors to strongly express the FSH receptor using IHC [35]. Compared to mucinous carcinomas, higher levels of FSH receptor DNA expression were noted in granulosa cell tumors in one study. Scattergram analysis by Chu, Rushdi [25] confirmed the expression of the FSH receptor to be significantly higher in granulosa cell tumors than epithelial tumors or normal ovaries.

#### 3.4.4. All Types of Ovarian Tumors

The differing types of ovarian tumors showed varying results, as summarized in Figure 3. Results of the studies provided various outcomes since different analyzing techniques were applied; the number of studies detecting (yes or no) expression of the FSH receptor and the percentages of positive tumors within the subgroups of ovarian tumors analyzed with IHC.

The highest mean percentages of FSH receptor-positive tumors shared a level of 59–60% for serous, endometrioid and sex cord–stromal tumors using IHC. Clear cell carcinomas had slightly lower rates, and other tumors had substantially lower rates of percentages. However, a greater disparity was observed in studies reporting expression by yes or no. FSH receptor expression was reported in all studies examining sex cord–stromal tumors. In the other carcinomas, the studies examining FSH receptor expression were less conclusive.

#### 3.4.5. Cell Line Studies

**Figure 4:**
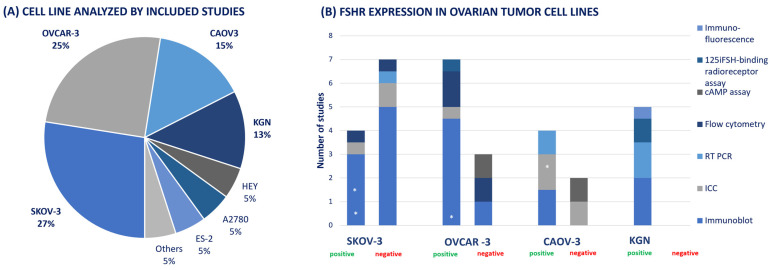
FSH receptor expression analyzed in ovarian tumor cell lines. (**A**) Studies examining FSH receptor in tumor cell lines (n = 23); (**B**) FSH receptor expression in four most examined ovarian tumor cell lines (SKOV-3, OVCAR-3, CAOV-3 and KGN) examined by various analyzing methods: immunofluorescence, [^125^I]-FSH-binding radioreceptor assay, cAMP assay, flow cytometry, RT-PCR, ICC and Immunoblot. * Low expression. ICC: immunocytochemistry; RT-PCR: reverse transcriptase polymerase chain reaction.

In addition to the tumor tissues examined, 23 articles examined the FSH receptor expression on ovarian tumor cell lines. Multiple cell lines were studied; the SKOV-3, OVCAR-3, CAOV-3 and KGN cell lines were analyzed most often, as visualized in Figure 4A. The cell lines originated from various types of ovarian tumors: the SKOV-3 cell line was derived from a serous cystadenocarcinoma, the OVCAR-3, CAOV-3 and HEY from high serous grade adenocarcinoma, and the KGN cell was a FOXL2-positive cell line derived from a granulosa cell tumor [40,43,44,45]. The remaining A2780 cell line originated from an endometrioid carcinoma and the ES-2 from a clear cell adenocarcinoma of the ovary.

Figure 4B shows the results of the FSH receptor presence in the four ovarian tumor cell lines mostly studied, examined by the following differing detection techniques: immunoblot, ICC, RT-PCR, flow cytometry, cAMP assay, [^125^I]-FSH-binding radioreceptor assay and immunofluorescence.

Of all studies conducting research on the SKOV-3 cell line, four showed positive results by detecting the FSH receptor with three different techniques, of which two reported low levels of expression [46,47,48,49]. Negative results were shown by seven studies, with four differing techniques [50,51,52,53,54,55,56]. The OVCAR-3 cell line showed positive results in seven studies using four techniques, of which one reported low levels of expression [46,47,50,52,53,56,57]. The three studies gaining negative results used three differing techniques [22,55,58]. Of the CAOV-3 cell line, four studies detected positive FSH receptor expression results with three techniques, one reporting low levels of these expressions [50,52,55,56]. The two studies that showed negative results used two techniques [22,58]. All of the five studies examining the KGN cell line using three varying methods reported positive FSH receptor expression [22,35,40,59,60]. No studies were found to be reporting negative results.

## 4. Discussion

### 4.1. Summary of Main Results

With this systematic review, we provide an overview of the existing literature regarding the expression of the FSH receptor in all types of ovarian cancer and explore its potential application in emerging theranostics as a treatment option. In the majority of ovarian tumor types, conflicting results were found regarding the presence of the FSH receptor, with the exception of sex cord–stromal tumors. In all 11 included articles conducting research on sex cord–stromal tumors, in particular, the most commonly studied granulosa cell tumors, the FSH receptor was detected through (RT) PCR, [^125^I]FSH-receptor-binding assay and IHC.

In cell line studies, serous cell lines showed inconsistent results, and the KGN cell line, derived from a granulosa cell tumor, was the only cell line to consistently show FSH receptor expression. Therefore, the available literature shows sex cord–stromal tumors to be the sole ovarian tumors to consistently express the FSH receptor.

### 4.2. Results in Context of Published Literature

Our findings point out that the expression of the FSH receptor is mainly found in the GCT, the most studied SCST. These results are consistent with other published literature since the FSH receptor is primarily present in the female ovarian in sex cord–stromal cells, in particular, the granulosa cells, where the FSH receptor is vital in the development of the reproduction system via FSH and later as a regulator for reproduction and the production of ova cells [10,61]. In addition, in the cell line studies, the GCT represented by the KGN cell line showed the presence of the FSH receptor. Multiple other sex cord–stromal tumor cell lines were established; however, none were reported to express functional FSH receptors, according to Nishi, Yanase [40].

Zheng, Magid [10] demonstrate FSH receptor expression in the ovarian surface epithelium and fallopian tube epithelium. This is in line with the findings of our review, where the included studies on epithelial subtypes, serous carcinomas specifically, observed this expression to be present prior to cell dedifferentiation and tumor progression. Thereby, since serous tumors often occur in late stages, varying results regarding the presence of the FSH receptor emerged. Of interest, multiple reviews confirmed the lack of expression of the FSH receptor in healthy tissue, in contrast to its correlation with the varying malignant tissue. For example, Radu, Pichon [11] discovered the FSH receptor to not be expressed in normal tissue located more than 10 mm from multiple tumors studied, including ovarian carcinomas, thereby supporting the hypothesis of applying theranostics targeting the FSH receptor to solely attack malignant tissue [11,12,13]. In addition, the FSH receptor appears to be involved in tumor angiogenesis in a variety of other cancer types, highlighting the value of using theranostics—entering through vascularization—to target the FSH receptor [11].

In conclusion, the FSH receptor is limited in females to the ovarian, where expression is found in epithelial as well as sex cord and stromal cells. However, during cell dedifferentiation, the FSH receptor appears to diminish in epithelial cells, giving varying results of expression in these epithelial carcinomas. After tumor progression, FSH receptor expression is found only consistently present in sex cord–stromal tumors.

### 4.3. Strengths and Weaknesses

This review includes all available information regarding the expression of the FSH receptor in every type of ovarian tumor, which forms the strength of this study. However, to gather all available literature, the publication date was not limited; thus, outdated articles may have been included. Dated articles form a limitation due to the use of obsolete techniques, making the results less reliable.

Second, these differences in techniques and heterogeneous outcomes of the included studies make it difficult to compare the various results. For example, one study reported a percentage, and the other study just confirmed the FSH receptor to be detected in the malignant tissue, whereby it is unclear to what extent the expression of the FSH receptor is. In addition, the most reliable technique is still to be discussed. In multiple reviews examining the FSH receptor, detection of the receptor was noted to be a challenge due to the lack of standard protocols regarding the most reliable technique. As a result, conflicting results were observed [12,13,15], as seen in the cell line studies where identical cell lines were examined by identical techniques; however, differing results emerged. These heterogenous results may be caused by differences in techniques and the lack of standard protocols.

### 4.4. Implications for Practice and Future Research

This overview of the literature has confirmed the hypothesis that the FSH receptor is indeed predominant in the subtype of sex cord–stromal tumors, in particular granulosa cell tumors. Given the natural origin of the FSH receptor on granulosa cells, it was expected that tumors of this origin would express the FSH receptor most frequently. Since expression in healthy tissue appears to be lacking, this receptor may function as a useful target for a new theranostic therapy that may be of help for patients with a granulosa cell tumor. Now that target selection is confirmed in the literature through this review, the development and validation of an FSH receptor-directed radiopharmaceutical is needed. If so, theranostics may be of value in patients suffering an SCST associated with recurrences and, thereby, reduced treatment options.

## 5. Conclusions

In conclusion, this systematic review demonstrates the expression of the FSH receptor in ovarian tumors with studies in sex cord–stromal tumors, particularly in granulosa cell tumors and the corresponding cell line KGN showing the most conclusive results on the presence of the receptor. The results of the studies regarding other subtypes of ovarian tumors yielded more inconsistent results in the tumor tissue as well as in the cell line studies. Confirmed expression of the FSH receptor allows this receptor to function as a possible target in the future theranostic approach in sex cord–stromal tumors.

## Figures and Tables

**Table 1:** Summary of studies examining FSH receptor expression in tumor tissue of subtypes of ovarian tumors; population (size and FIGO stage at diagnosis), analyzing methods used and corresponding results.

Author (Year)	Patients (n)	FIGO Stage at Diagnosis	Analyzing Methods	FSH Receptor Expression (%)
		(% of Total)		
Serous tumors				
Cheung (2020) [22]	112	II (1.8)	IHC	65.2
		III (91.1)		
		IV (7.1)		
Lenhard (2011) [23]	110	I (22.6)	IHC	64.5
		II (5.8)		
		III (70.3)		
		IV (1.3)		
Garrido (2020) [24]	NA	NA	RT-PCR on RNA	Yes ^1^
			IHC	
Chu (2002) [25]	9	IIIC (88.9)	RT-PCR on RNA	Little—no ^2^
		NA (11.1)		
Wang (2003) [26]	17	I (35.3)	RT-PCR on RNA	Yes ^1^
		II (35.3)		
		III (29.4)		
Minegishi (2000) [27]	9	Ia (16.6) ^3^	Immunoblot	0
		2c (5.6)	RT-PCR on RNA	22.2
		IIIa (11.1)		
		IIIb (5.6)		
		IIIc (38.9)		
		IV (22.2)		
Perales (2017) [14]	28	III/IV	Immunoblot	50
			IHC	
Nakano (1989) [28]	7	NA	^125^IFSH-binding radioreceptor assay	No ^2^
Zheng (2000) [29]	17	NA	RT-PCR on RNA	59
			ISH	65
			IHC	53
Stouffer (1984) [30]	13	NA	^125^IFSH-binding radioreceptor assay	No ^2^
High-grade serous tumors				
Cheung (2020) [22]	29	I (13.8)	RT-PCR on RNA	Little—no ^2^
		II (27.6)		
		III (58.6)		
Feng (2016) [31]	863 ^4^	I and II (9)	IHC	55%
		III and IV (91)		
Feng (2017) [32]	214	I and II (3.7)	IHC	44.9 (P ^5^)
		III and IV (96.3)		55.3 (R ^6^)
Low-grade serous tumors				
Feng (2017) [33]	25	I (23.1)	IHC	84
		III (73.1)		
		IV (3.8)		
Mucinous tumors				
Lenhard (2011) [23]	21	I (22.6) ^3^	IHC	61.9
		II (5.8)		
		III (70.3)		
		IV (1.3)		
Burger (1998) [34]	7	NA	RT-PCR on RNA	Yes ^1^
Chu (2002) [25]	8	NA (1)	RT-PCR on RNA	No ^2^
		Benigne (1)		
		I (5)		
		II (1)		
Zheng (2000) [29]	4	I (10) ^3^	RT-PCR on RNA	50
		II (23.3)	ISH	50
		III (50)	IHC	25
		IV (16.7)		
Perales (2017) [14]	9	NA	Immunoblot	67
Minegishi (2000) [27]	3	Ia (16.6) ^7^		
		2c (5.6)		
		IIIa (11.1)	Immunoblot	33.3
		IIIb (5.6)	RT-PCR on RNA	100
		IIIc (38.9)		
		IV (22.2)		
Nakano (1989) [28]	2	NA	^125^IFSH-binding radioreceptor assay	No ^2^
Stouffer (1984) [30]	3	NA	^125^IFSH-binding radioreceptor assay	No ^2^
Adult granulosa cell tumors				
Haltia (2020) [35]	138 ^7^	I (91)	IHC	60
		II (6)		
		III (1)		
		NA (2)		
	10	NA	RT-PCR on RNA	Yes ^1^
Burger (1998) [34]	3	NA	RT-PCR on RNA	Yes ^1^
Chu (2002) [25]	7	I (42.9)	RT-PCR on RNA	Yes ^1^
		IA (14.3)		
		R ^6^ (28.6)		
		NA (14.3)		
Giacigla (2000) [36]	89	NA	RT-PCR on RNA	Yes ^1^
Fuller (1998) [37]	3	I (100)	RT-PCR on RNA	Yes ^1^
	129	Ia (16.7)	PCR op DNA	Yes ^1^
		I (16.7)		
		IIc (8.3)		
		III (16.7)		
		NA (16.6))		
McNeilage (2007) [38]	1	NA ^8^	RT-PCR on RNA	Yes ^1^
Reinholtz (2000) [39]	12	I (58.3)	RT-PCR on RNA	Yes ^1^
		III (8.4)		
		IV (33.3)		
Nishi (2001) [40]	1	III (8.4)	^125^IFSH-binding radioreceptor assay	Yes ^1^
Nakano (1989) [28]	1	NA	^125^IFSH-binding radioreceptor assay	Yes ^1^
Sertoli–Leydig cell tumors				
Choong (2002) [41]	1	I (100)	RT-PCR on RNA	Yes ^1^
Granulosa theca cell tumor				
Stouffer (1984) [30]	1	NA	^125^IFSH-binding radioreceptor assay	Yes ^1^
Endometrioid tumors				
Lenhard (2011) [23]	12	I (22.6) ^3^	IHC	58.3
		II (5.8)		
		III (70.3)		
		IV (1.3)		
Zheng (2000) [29]	2	I (10) ^3^	IHC	50
		II (23.3)	RT-PCR on RNA	86
		III (50)	ISH	50
		IV (16.7)		
Minegishi (2000) [27]	4	Ia (16.6) ^3^		
		2c (5.6)		
		IIIa (11.1)	RT-PCR on RNA	50
		IIIb (5.6)	immunoblot	0
		IIIc (38.9)		
		IV (22.2)		
Perales (2017) [14]	23	NA	IHC	70
			immunoblot	70
Stouffer (1984) [30]	1	NA	^125^IFSH-binding radioreceptor assay	No ^2^
Clear cell tumors				
Lenhard (2011) [23]	13	NA	IHC	61.5
Zheng (2000) [29]	3	I (10) ^3^	IHC	67
		II (23.3)	RT-PCR on RNA	67
		III (50)	ISH	67
		IV (16.7)		
Minegishi (2000) [27]	2	Ia (16.6) ^3^		
		2c (5.6)		
		IIIa (11.1)	RT-PCR on RNA	50
		IIIb (5.6)	immunoblot	0
		IIIc (38.9)		
		IV (22.2)		
Perales (2017) [14]	12	NA	IHC	33
			immunoblot	33
Germ cell tumors				
Nakano (1989) [28]	4	NA	^125^IFSH-binding radioreceptor assay	No ^2^
Stouffer (1984) [30]	1	NA	^125^IFSH-binding radioreceptor assay	No ^2^
Brenner tumors				
Nakano (1989) [28]	2	NA	^125^IFSH-binding radioreceptor assay	No ^2^
Carcinosarcoma NOS				
Perales (2017) [14]	4	NA	Western blotting	0
Carcinoma, undifferentiated, NOS				
Stouffer (1984) [30]	3	NA	^125^IFSH-binding radioreceptor assay	No ^2^

^1^ FSH receptor expression was detected; ^2^ FSH receptor expression was not detected; ^3^ reported stages are of all ovarian tumor types examined in the article, stage per tumor type unclear; ^4^ tissue microarrays (TMA); ^5^ primary tumors; ^6^ recurrent tumors; ^7^ 175 formalin-fixed paraffin-embedded samples; ^8^ the patient was not formally staged as there was no obvious macroscopic disease seen elsewhere in the abdomen at the time of her initial surgery. IHC: immunohistochemistry; RT: reverse transcriptase; PCR: polymerase chain reaction; [^125^I]FSH: Iodium-labeled FSH.

## Data Availability

The data presented in this study are available on request from the corresponding author.

## Supplementary Materials

The following supporting information can be downloaded at https://www.mdpi.com/article/10.3390/cancers16061140/s1, Supplementary Text S1: Systematic search strings; Supplementary Figure S1: Risk-of-bias assessments. (a) Quality assessment of the included descriptive cross sectional studies using the JBI tool. (b) Quality assessment of the included case report studies using the Murads tool. References [14,22,23,24,25,26,27,28,29,30,31,32,33,34,35,36,37,38,39,40,41,46,47,48,49,50,51,52,53,54,55,56,57,58,59,60,62,63,64,65,66] are cited in the Supplementary Materials.
