# Supplementary material for: Follicle-Stimulating Hormone Receptor Expression and Its Potential Application for Theranostics in Subtypes of Ovarian Tumors: A Systematic Review
Source: Cancers (Basel). 2024 Mar 13;16(6):1140. doi: 10.3390/cancers16061140 (PMC10969425; doi:10.3390/cancers16061140)
Supplement: Supplementary file 1 [file cancers-16-01140-s001.zip › cancers-2876022-supplementary.pdf]

Supplementary Text S1

Systematic search strings

Pubmed (466 records – 23/01/2024)

((("Ovary"[MeSH Terms] OR "ovar*"[Title/Abstract] OR "gonad*"[Title/Abstract]) AND ("Neoplasms"[MeSH Terms] OR "tumor*"[Title/Abstract] OR "tumour*"[Title/Abstract] OR "neoplasm*"[Title/Abstract] OR "carcinom*"[Title/Abstract] OR "cancer*"[Title/Abstract] OR "sarcom*"[Title/Abstract] OR "cystadenom*"[Title/Abstract] OR "papillom*"[Title/Abstract] OR "adenofibroma*"[Title/Abstract] OR "cystadenofibroma*"[Title/Abstract] OR "adenocarcinom*"[Title/Abstract] OR "adenom*"[Title/Abstract])) OR (("epithelial*"[Title/Abstract] OR "serous*"[Title/Abstract] OR "brenner*"[Title/Abstract] OR "clear cell*"[Title/Abstract] OR "seromucinous*"[Title/Abstract] OR "endometrioid*"[Title/Abstract] OR "mucinous*"[Title/Abstract] OR "mesenchym*"[Title/Abstract] OR "germ cell*"[Title/Abstract] OR "miscellaneous*"[Title/Abstract] OR "mesonephric*"[Title/Abstract] OR "undifferentiated*"[Title/Abstract] OR "dedifferentiated*"[Title/Abstract] OR "mixed cell*"[Title/Abstract] OR "smooth muscle*"[Title/Abstract] OR "mixed epithelial and mesenchymal*"[Title/Abstract] OR "germ cell*"[Title/Abstract] OR "yolk sac*"[Title/Abstract] OR "embryonal*"[Title/Abstract] OR "chorio*"[Title/Abstract] OR "mixed germ cell*"[Title/Abstract] OR "struma ovary*"[Title/Abstract] OR "stromal*"[Title/Abstract] OR "germ cell sex cord stromal*"[Title/Abstract] OR "germ cell sex cord stromal*"[Title/Abstract] OR "gonadoblastom*"[Title/Abstract] OR "undifferentiated gonadal tissue*"[Title/Abstract] OR "miscellaneous*"[Title/Abstract] OR "rete ovar*"[Title/Abstract] OR "wolff*"[Title/Abstract] OR "solid pseudopapill*"[Title/Abstract] OR "small cell*"[Title/Abstract] OR "wilms*"[Title/Abstract]) AND ("Neoplasms"[MeSH Terms] OR "tumor*"[Title/Abstract] OR "tumour*"[Title/Abstract] OR "neoplasm*"[Title/Abstract] OR "carcinom*"[Title/Abstract] OR "cancer*"[Title/Abstract] OR "sarcom*"[Title/Abstract] OR "cystadenom*"[Title/Abstract] OR "papillom*"[Title/Abstract] OR "adenofibroma*"[Title/Abstract] OR "cystadenofibroma*"[Title/Abstract] OR "adenocarcinom*"[Title/Abstract] OR "adenom*"[Title/Abstract])) OR ("adenosarcom*"[Title/Abstract] OR "myxoma*"[Title/Abstract] OR "leiomyo*"[Title/Abstract] OR "carcinosarcom*"[Title/Abstract] OR "teratom*"[Title/Abstract] OR "dysgerminom*"[Title/Abstract] OR "gonadoblast*"[Title/Abstract]))

AND

("receptors, fsh"[MeSH Terms] OR "follicle stimulating hormone receptor*"[Title/Abstract] OR "FSHR"[Title/Abstract] OR "fsh receptor*"[Title/Abstract])

Embase (249 records – 23/01/2024)

Filter:

- Sources: Embase, Embase+MEDLINE
- Publication types: article, article in press

((('ovary'/exp OR 'ovar*':ti,ab,kw OR 'gonad*':ti,ab,kw) AND ('neoplasm'/exp OR 'tumor*':ti,ab,kw OR 'tumour*':ti,ab,kw OR 'neoplasm*':ti,ab,kw OR 'carcinom*':ti,ab,kw OR 'cancer*':ti,ab,kw OR 'sarcom*':ti,ab,kw OR 'cystadenom*':ti,ab,kw OR 'papillom*':ti,ab,kw OR 'adenofibroma*':ti,ab,kw OR 'cystadenofibroma*':ti,ab,kw OR 'adenocarcinom*':ti,ab,kw OR 'adenom*':ti,ab,kw)) OR (('epithelial*':ti,ab,kw OR 'serous*':ti,ab,kw OR 'brenner*':ti,ab,kw OR 'clear cell*':ti,ab,kw OR 'seromucinous*':ti,ab,kw OR 'endometrioid*':ti,ab,kw OR 'mucinous*':ti,ab,kw OR 'mesenchym*':ti,ab,kw OR 'germ cell*':ti,ab,kw OR 'miscellaneous*':ti,ab,kw OR 'mesonephric*':ti,ab,kw OR 'undifferentiated*':ti,ab,kw OR 'dedifferentiated*':ti,ab,kw OR 'mixed cell*':ti,ab,kw OR 'smooth muscle*':ti,ab,kw OR 'mixed epithelial and mesenchymal*':ti,ab,kw OR 'germ cell*':ti,ab,kw OR 'yolk sac*':ti,ab,kw OR 'embryonal*':ti,ab,kw OR 'chorio*':ti,ab,kw OR 'mixed germ cell*':ti,ab,kw OR 'struma ovary*':ti,ab,kw OR 'stromal*':ti,ab,kw OR 'germ cell sex cord stromal*':ti,ab,kw OR 'germ cell sex cord stromal*':ti,ab,kw OR 'gonadoblastom*':ti,ab,kw OR

‘undifferentiated gonadal tissue*’:ti,ab,kw OR ‘miscellaneous*’:ti,ab,kw OR ‘rete ovar*’:ti,ab,kw OR ‘wolff*’:ti,ab,kw OR ‘solid pseudopapill*’:ti,ab,kw OR ‘small cell*’:ti,ab,kw OR ‘wilms*’:ti,ab,kw) AND ('neoplasm'/exp OR ‘tumor*’:ti,ab,kw OR ‘tumour*’:ti,ab,kw OR ‘neoplasm*’:ti,ab,kw OR ‘carcinom*’:ti,ab,kw OR ‘cancer*’:ti,ab,kw OR ‘sarcom*’:ti,ab,kw OR ‘cystadenom*’:ti,ab,kw OR ‘papillom*’:ti,ab,kw OR ‘adenofibroma*’:ti,ab,kw OR ‘cystadenofibroma*’:ti,ab,kw OR ‘adenocarcinom*’:ti,ab,kw OR ‘adenom*’:ti,ab,kw)) OR (‘adenosarcom*’:ti,ab,kw OR ‘myxoma*’:ti,ab,kw OR ‘leiomyo*’:ti,ab,kw OR ‘carcinosarcom*’:ti,ab,kw OR ‘teratom*’:ti,ab,kw OR ‘dysgerminom*’:ti,ab,kw OR ‘gonadoblast*’:ti,ab,kw))

AND

('follitropin receptor'/exp OR ‘follicle stimulating hormone receptor*’:ti,ab,kw OR ‘FSHR’:ti,ab,kw OR ‘fsh receptor*’:ti,ab,kw)

Cochrane (5 records – 23/01/2024)

(((ovar*:ti,ab,kw OR gonad*:ti,ab,kw) AND (tumor*:ti,ab,kw OR tumour*:ti,ab,kw OR neoplasm*:ti,ab,kw OR carcinom*:ti,ab,kw OR cancer*:ti,ab,kw OR sarcom*:ti,ab,kw OR cystadenom*:ti,ab,kw OR papillom*:ti,ab,kw OR adenofibroma*:ti,ab,kw OR cystadenofibroma*:ti,ab,kw OR adenocarcinom*:ti,ab,kw OR adenom*:ti,ab,kw)) OR ((epithelial*:ti,ab,kw OR serous*:ti,ab,kw OR brenner*:ti,ab,kw OR “clear” NEXT cell*:ti,ab,kw OR seromucinous*:ti,ab,kw OR endometrioid*:ti,ab,kw OR mucinous*:ti,ab,kw OR mesenchym*:ti,ab,kw OR “germ” NEXT cell*:ti,ab,kw OR miscellaneous*:ti,ab,kw OR mesonephric*:ti,ab,kw OR undifferentiated*:ti,ab,kw OR dedifferentiated*:ti,ab,kw OR “mixed” NEXT cell*:ti,ab,kw OR “smooth” NEXT muscle*:ti,ab,kw OR “mixed epithelial and” NEXT mesenchymal*:ti,ab,kw OR “germ” NEXT cell*:ti,ab,kw OR “yolk” NEXT sac*:ti,ab,kw OR embryonal*:ti,ab,kw OR chorio*:ti,ab,kw OR “mixed germ” NEXT cell*:ti,ab,kw OR “struma” NEXT ovary*:ti,ab,kw OR stromal*:ti,ab,kw OR “germ cell sex cord” NEXT stromal*:ti,ab,kw OR “germ cell sex cord” NEXT stromal*:ti,ab,kw OR gonadoblastom*:ti,ab,kw OR “undifferentiated gonadal” NEXT tissue*:ti,ab,kw OR miscellaneous*:ti,ab,kw OR “rete” NEXT ovar*:ti,ab,kw OR wolff*:ti,ab,kw OR “solid” NEXT pseudopapill*:ti,ab,kw OR “small” NEXT cell*:ti,ab,kw OR wilms*:ti,ab,kw) AND (tumor*:ti,ab,kw OR tumour*:ti,ab,kw OR neoplasm*:ti,ab,kw OR carcinom*:ti,ab,kw OR cancer*:ti,ab,kw OR sarcom*:ti,ab,kw OR cystadenom*:ti,ab,kw OR papillom*:ti,ab,kw OR adenofibroma*:ti,ab,kw OR cystadenofibroma*:ti,ab,kw OR adenocarcinom*:ti,ab,kw OR adenom*:ti,ab,kw)) OR (adenosarcom*:ti,ab,kw OR myxoma*:ti,ab,kw OR leiomyo*:ti,ab,kw OR carcinosarcom*:ti,ab,kw OR teratom*:ti,ab,kw OR dysgerminom*:ti,ab,kw OR gonadoblast*:ti,ab,kw))

AND

(“follicle stimulating hormone” NEXT receptor*:ti,ab,kw OR FSHR*:ti,ab,kw OR “fsh” NEXT receptor*:ti,ab,kw)

Supplementary Figure S1

Risk of bias assessments

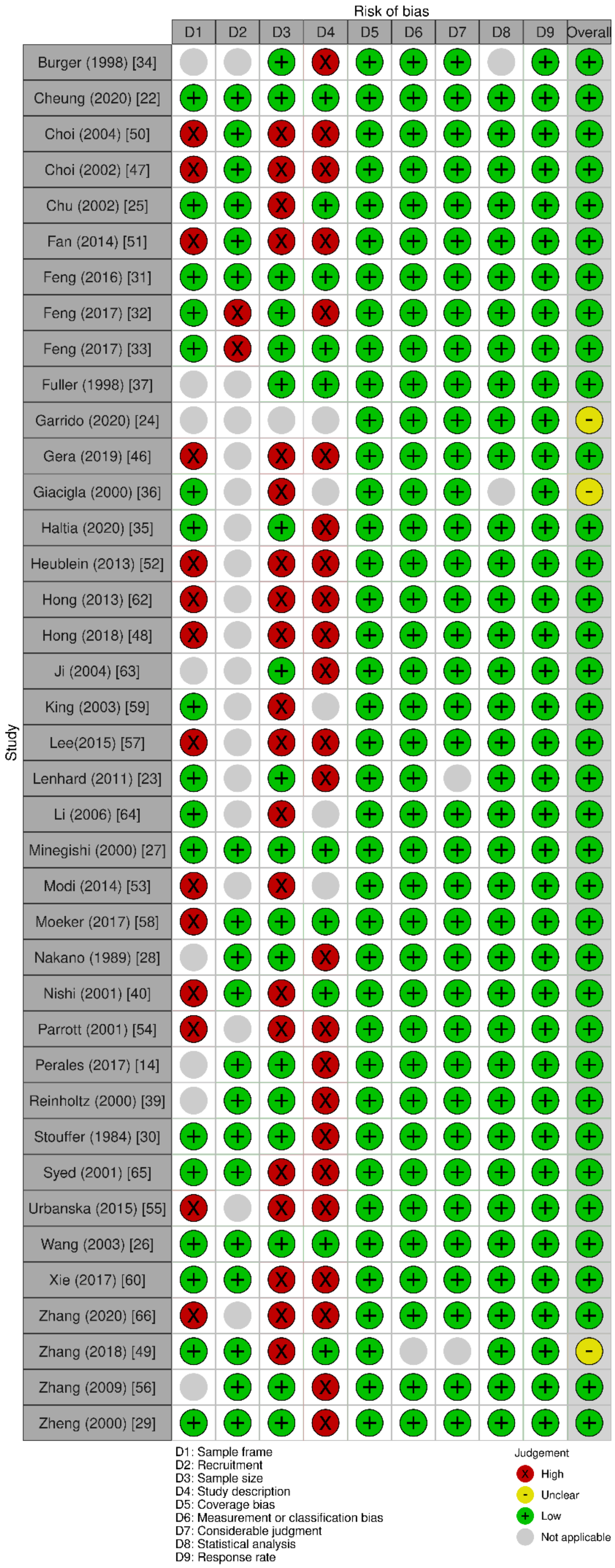

(a)

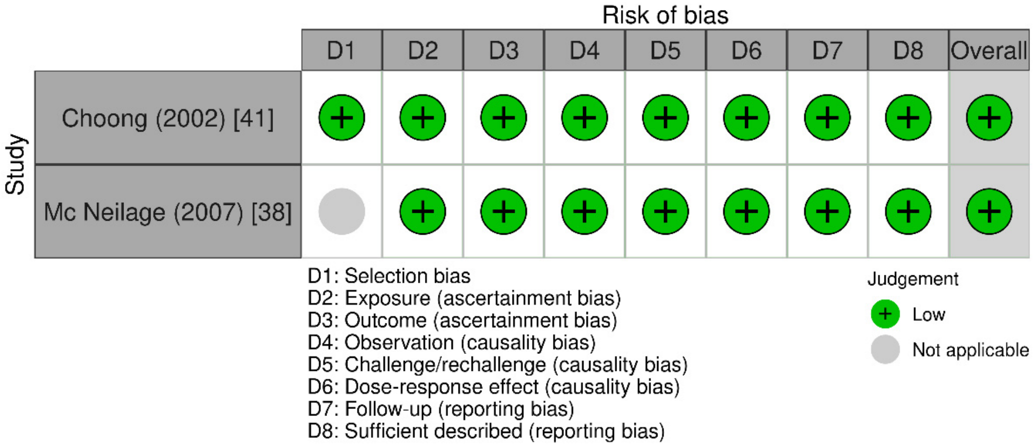

(b)

Figure S1. (a) Quality assessment of the included descriptive cross sectional studies using the JBI tool. (b) Quality assessment of the included case report studies using the Murads tool.
